# Mating-dependent lifespan cost of sterol depletion in male *Drosophila melanogaster*

**DOI:** 10.1073/pnas.2533735123

**Published:** 2026-06-02

**Authors:** Andrew W. McCracken, Nicola White, Jola Tanianis-Hughes, Elena Rosca, Thomas Gill, Libby McKinney, Stephen J. Simpson, Klaus Reinhardt, Marko Brankatschk, Matthew D. W. Piper, Juliano Morimoto, Stuart Wigby

**Affiliations:** ^a^ Department of Evolution, Ecology and Behaviour, Institute of Infection, Veterinary and Ecological Sciences, Faculty of Health and Life Sciences, University of Liverpool, Liverpool CH64 7TE, United Kingdom; ^b^ Charles Perkins Centre and School of Life and Environmental Sciences, University of Sydney, Sydney, New South Wales 2006, Australia; ^c^ Applied Zoology, Faculty of Biology, Technische Universität Dresden, Dresden 01069, Germany; ^d^ Plant Cell and Molecular Biology, Biotechnology Center, Center for Molecular and Cellular Bioengineering, Technische Universität Dresden, Dresden 01217, Germany; ^e^ Faculty of Science, School of Biological Sciences, Monash University, Clayton, Victoria 3800, Australia; ^f^ School of Natural and Computing Science, Institute of Mathematics, School of Natural and Computing Sciences, University of Aberdeen, Aberdeen AB24 5PZ, United Kingdom; ^g^Setor de Ciências Biológicas, Programa de Pós-Graduação em Ecologia e Conservação, Universidade Federal do Paraná, Curitiba, Paraná 81531-980, Brazil

**Keywords:** nutrition, ageing, diet, reproduction, *Drosophila*

## Abstract

Trade-offs between lifespan and reproduction are a central theme in evolution and ecology, often attributed to physiological constraints on the allocation of limiting resources. However, recent work suggests such trade-offs may depend on macro- and micronutritional context. We examined how macronutrient balance and cholesterol availability shape lifespan and reproductive performance in male *Drosophila melanogaster*, explicitly testing both unmated males and those permitted to mate freely throughout life—a design rarely applied. We found that cholesterol deprivation substantially reduced lifespan, but only in frequently mating males, indicating that sterols play a key role in mediating the lifespan costs of mating, consistent with their known role in sperm and seminal fluid. Conversely, the inclusion of dietary cholesterol reduced lifespan in unmated males maintained on low protein-to-carbohydrate diets, suggesting that male reproductive investment is sufficiently high to generate sterol-dependent costs to the soma. Lifespan was maximized at higher protein-to-carbohydrate ratios than previously estimated, while reproductive performance in aged males was favored by carbohydrate-biased diets, demonstrating a nutritional trade-off. Males preferentially consumed macronutrient ratios that promoted reproductive success rather than longevity and discriminated the presence of cholesterol in food. Together, these findings reveal sterols as a key mediator of male longevity, with effects that depend critically on both diet and reproductive context. More broadly, by integrating sterols, macronutrient balance, and sustained reproductive investment, our study adds to the growing evidence that male reproductive costs can be substantial and refines our understanding of the evolutionary basis of dietary trade-offs.

## Significance

Male reproduction can impose substantial costs, but how these costs depend on nutrient context remains unclear. Using male fruit flies on chemically defined diets, we show that removing dietary sterols shortens lifespan in mating males, whereas unmated males sometimes experience reduced lifespan when cholesterol is present, indicating costs of overconsumption. These opposing effects suggest that sterols mediate lifespan in a context-dependent manner. We also identify a clear nutritional trade-off, with diets that maximize lifespan differing from those that maximize late-life reproduction. These findings reveal that male aging is strongly shaped by nutrient interactions, not just energy intake.

Organisms exhibit remarkable plasticity in response to the nutritional environment, adjusting life-history traits such as lifespan and reproductive output in response to dietary availability and quality (1). Within nutritional ecology, a central question concerns how and why such traits are shaped by nutrition, especially when these traits appear to trade-off with one another; known as a “nutritional trade-off” (2–4). The evolutionary basis of these nutritional trade-offs—particularly how organisms allocate limited nutritional resources to maximize fitness—remains a key focus. At present, where nutritional trade-offs are observed (where fitness traits vary in their nutritional optima) this is sometimes interpreted as evidence for a physiological trade-off, where energy derived from nutrients are allocated toward disparate strategies (5). Should physiological trade-offs indeed represent the primary drivers of dietary-mediated lifespan plasticity, this would imply biological constraints on our ability to extend lifespan via dietary manipulations without inhibiting reproduction.

Much of our mechanistic understanding of nutritional effects on life-history traits stems from studies in model organisms, where experimental control permits detailed manipulation of diet. Research has traditionally focused on the macronutrient composition of the diet—specifically the relative balance of protein and carbohydrate—as these have been shown to be a major driver of variation in lifespan and reproductive success (6–9). More recently, attention has turned to finer scale dietary components, such as amino acids (10, 11), and micronutrients, like sterols, (12–14), which can exert substantial effects on life-history traits even absent of energetic contributions. Sterols, in particular, are of interest in insects, which are auxotrophic for these compounds and must acquire them from the diet (15). These micronutrients have evolutionarily conserved roles and are essential for membrane stability and hormone production (16) and may thus play underappreciated roles in shaping life-history trade-offs.

Emerging work suggests that previously observed trade-offs—especially in females—may not reflect physiological constraints, but instead result from dietary imbalances. For example, diets precisely matched to the exome-derived amino acid requirements of the organism (11), or depleted of sufficient dietary cholesterol (13), can obscure otherwise strong trade-offs between lifespan and reproduction. However, while females often exhibit a nutritional trade-off between these traits (6, 7, 17), previous studies have reported that male *D. melanogaster* (18, 19), and field crickets (*Teleogryllus commodus*) (8) appear to simultaneously maximize both lifespan and reproduction under low protein-to-carbohydrate (P:C) conditions, and that their reproductive performance is invariant to dietary sterol levels (12). This raises important questions about the generality of nutritional trade-offs across sexes. Notably, male reproductive investment may be contingent on access to mating opportunities; unlike females, whose reproductive investment can proceed long after a single mating event, males require ongoing access to mates to invest in energetically costly ejaculate production (20). As most dietary experiments house males in single-sex groups for most of their lives, true reproductive investment is likely underestimated. If sterols are functionally important for ejaculate quality, the consequences of sterol manipulation may only manifest under conditions of sustained reproductive effort. The apparent absence of a male trade-off may thus reflect limitations in experimental design rather than biological reality. A nonexclusive alternative is that sex-specific nutritional requirements compress male intake targets: Female reproduction typically demands higher protein for oogenesis, whereas male reproductive effort is relatively more carbohydrate-dependent. Consequently, male optima for lifespan and reproduction may lie closer together than in females (8, 17), making male trade-offs harder to detect in males.

To address these fundamental problems, we used *Drosophila melanogaster* to test whether a nutritional trade-off between lifespan and reproduction exists in males while manipulating key, often overlooked factors. By conducting experiments in both single-sex and mixed-sex environments, we were able to test whether mating opportunity alters the expression of nutritional trade-offs in males. Specifically, we used unmated males and males maintained with continuous access to females as two biologically interpretable endpoints, capturing the presence, and absence of, sustained reproductive effort. This design allowed us to test whether the effects of dietary sterols on lifespan are contingent on prolonged reproductive activity, rather than attempting to replicate naturalistic mating frequencies. We measured both lifespan and a range of metrics associated with pre- and postcopulatory reproductive success in young and old males, to capture the age-specific consequences of diet. Further, we employed a fully chemically defined (holidic) solid diet (21), in which the amino acid composition was matched to the fly’s exome profile (11), providing precise control over dietary components. This approach overcomes the limitations of traditional yeast-based diets, which are compositionally complex and contain multiple confounding bioactive compounds (22). The solid format further avoids potential artifacts associated with liquid feeding assays (CAFE), which can suppress feeding and impair fitness (*SI Appendix*, Table S1). In addition to manipulating the P:C ratio, we introduced a third nutritional dimension by varying the presence or absence of dietary sterols.

Based on previous literature, we predicted that 1) male reproductive success would decline with age; 2) unmated males would exhibit greater longevity and higher late-life reproductive potential than mated males; 3) males would maximize both lifespan and reproduction at low P:C ratios; 4) the addition or depletion of dietary sterols might modify P:C optima, particularly under conditions of sustained reproductive investment due to the potential lack of sterols in sperm and seminal fluid.

## Materials and Methods

### Fly Husbandry.

Focal males were wild-type Dahomey (*Dah*) (23), an outbred stock originally acquired in 1970 from Benin. Scarlet (*st^1^*) (24) recessive eye mutant females were used for focal mating experiments; males were additionally used for remating experiments to allow the paternity assignment of resulting offspring (25). *st^1^* had previously been serially backcrossed into the Dahomey genetic background (25). To easily differentiate between males and females in mating treatments, *white* Dahomey females (w^Dah^) (26) were used to permit focal males ad libitum mating opportunities. All genotypes were maintained prior to experiments at 25C, on SY media (10% m/v yeast, 10% m/v sucrose, 2% m/v agar, 0.3% v/v propionic acid, and 0.3 % m/v nipagin) in a mix of bottles and large population cages, to maintain genetic diversity. For growing experimental flies, we used the standard larval density method (27). At eclosion, virgins were sorted under light CO2 anesthesia (<5 mins; <5L/min; Flowbuddy, SLS); experimental males were placed onto their experimental diet within 6 h of eclosion. All experimental diets for adult flies used were fully holidic, as in (21), with 21.4 g/L (200 N) amino acid concentration used as a standard. Dietary amino acids comprised of a *D. melanogaster* exome-matched amino acid ratio (Flyaa) (11). Relative dietary component concentration was kept in line with this template with the exception of sucrose, which was increased slightly to maintain an equal contribution of protein and carbohydrates (Dataset S1).

### Lifespan Experiments.

Focal males were provided with ad libitum access to solid media and maintained at 25C under a 12:12 light/dark cycle. Frequently mated males (FM) were maintained in at least a 2:1 female:male ratio, while unmated males (UM) were housed in single-sex groups. These treatments represent two biologically interpretable endpoints, contrasting the absence of mating with sustained reproductive effort throughout life. These extremes are designed to isolate the role of reproductive context, and permit the interpretation of diet x mating status interactions, rather than mimic naturalistic conditions. For FM treatments, w^Dah^ females were maintained on the same experimental holidic diets as focal males and were replenished every 14 d to prevent coaging effects. These females were distinct from those used in experiment 4 female assays. Fly sorting was staggered over the course of two days. Escapees, deaths due to extrinsic causes, and males used in reproductive assays were appropriately right-censored in survival analysis.

### Experiment 1: Nutritional Geometry (NG) Lifespan Experiment.

**Fig. 1.**
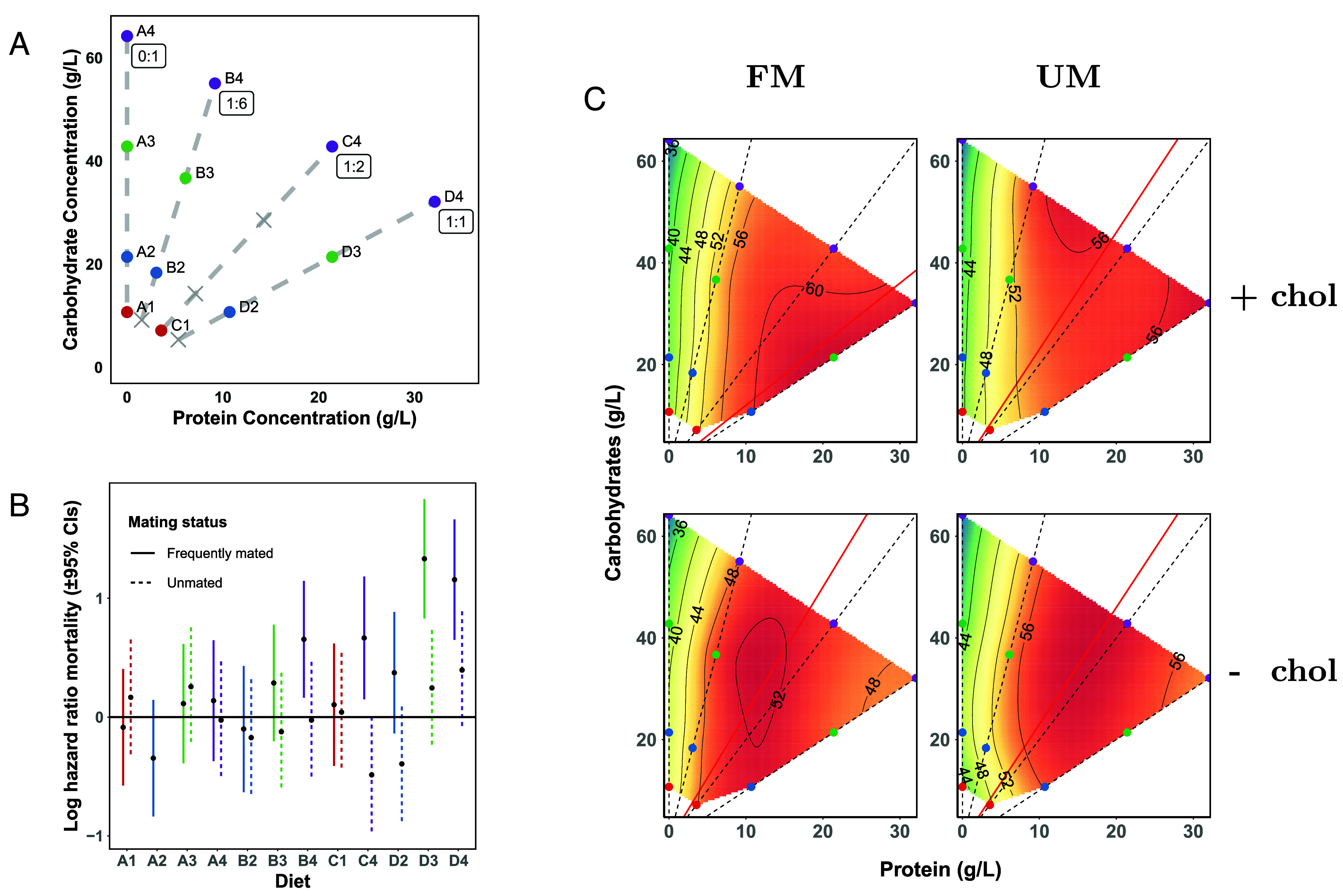
Dietary cholesterol deprivation causes mortality exacerbation exclusively in frequently mated males at higher protein-to-carbohydrate ratio (P:C) and nutrient concentrations. Lifespan optima exist at ~1:2 P:C. (*A*) Schematic of fixed diets used to generate nutritional landscapes of lifespan and to test the effect of cholesterol depletion. Diet B3 represents the low-protein (LP) diet in further experiments; D3 represents the high-protein (HP) diet. (*B*) Log hazard ratio mortality of the unique effect of dietary cholesterol deprivation, in frequently mated (FM) and unmated (UM) flies across diets shown in panel A. Values are expressed relative to cholesterol-containing diets (baseline = 0), with positive values indicating increased mortality when cholesterol is omitted from the diet. Condition A2 UM (-chol) was excluded due to anomalously high early mortality which was not observed in adjacent diet groups, and likely reflects experimental artifact. Increased mortality is observed in FM but not UM males across the nutritional landscape. N = 2,111 focal males. (*C*) Nutritional landscapes of male lifespan across protein-to-carbohydrate (P:C) ratios and nutrient concentrations, shown separately for FM and UM males, with and without cholesterol. Estimated optima for each treatment are indicated as red lines. Landscapes were fitted using a smoothing parameter of λ = 0.35. N = 1,465 focal males.

Twelve diets, over four P:C and four nutrient densities in a fractional factorial design (28) were used to cover the area of the landscape thought to be relevant to male fitness (18, 19, 29). For example, diet D3 (1:1 P:C; Fig. 1*A*) consisted of 21.4 g/L of both protein and carbohydrates. Our most nutritionally dense diets (i.e., A4, B4, C4, and D4; Fig. 1*A*) consisted of a nutritional concentration (protein + carbohydrates/volume) of 64.2 g/L. Topographical differences between both frequently mated (FM), and unmated (UM) males, and cholesterol-provided (chol) and cholesterol-deprived (no-chol) males were measured (Fig. 1*A* and Dataset S1). Performance landscapes were plotted by mapping lifespan responses to fixed ratio of P:C in the diet, rather than diet intake. Males were allocated to one diet, in the presence or absence of cholesterol (0 g/L or 0.3 g/L), and in the presence or absence of females (FM or UM). Males were housed in vials containing 4 ml media and maintained at a density of 15 flies per vial, (n = 45 focal males per P:C, cholesterol status, and mating status grouping). Flies were scored for mortality and provided fresh media 3 times a week.

To maximize food use and maintain a consistent density, focal males were consolidated across vials as densities declined over time; as a result, individuals were not maintained within consistent vial-level groupings throughout the experiment, precluding the use of vial as a random effect in subsequent analyses. In one condition (unmated A2O), 90% mortality occurred within a 4-d period of the assay, a pattern not observed in adjacent diet groups (A1, A3, A4). As this was inconsistent with typical mortality trajectories and likely reflects experimental artifact, this condition was excluded from analyses.

### Experiment 2: “Direct Comparison” High-Powered Lifespan and Reproduction Experiment.

#### Lifespan assays.

To test whether the costs to lifespan of sterol depletion arose in an age-dependent manner, or if sterol depletion mediated a concomitant cost to reproduction, we carried out a higher-powered, direct comparison among two P:C ratios chosen from the NG landscape generated in experiment 1. We chose two diets that we found to be close to the lifespan optima in our experiments: a relatively low protein diet (1:6 P:C; diet B3) and a relatively high protein diet (HP; 1:1 P:C; diet D3). For each P:C ratio we included both cholesterol-containing (0.3 g/L) (LPC and HPC) and cholesterol-absent (LPO and HPO) diets. As with experiment 1 (NG), FM or UM males were fully factorially allocated to one diet. Flies were housed in purpose-built demography cages (30) and maintained at a density of 120 flies per cage (n = 500 to 600 focal males per P:C, cholesterol status, and mating status grouping). Both available vials were allocated the same experimental diet containing 4 ml media. Flies were scored for mortality and provided fresh media every other day.

#### Male reproduction assays.

To test the effects of P:C manipulation and cholesterol depletion, we measured multiple parameters of male reproductive investment. Focal males at 1 wk and 5 wk old were removed from experimental cages in the direct comparison experiment under light anesthesia. Three days later we conducted mating trials. Reproductive assays followed a multistage design (*SI Appendix*, Fig. S1). After removal from the cages, males were individually housed on their experimental diet (n = ~75 focal males per P:C, cholesterol status, and mating status grouping). Virgin *st^1^*females were sorted concurrently and maintained in groups of 10. Females were provided with supplemental live yeast throughout, unless specified otherwise. Individual females were transferred into male vials on the day of the experiment, where focal male latency to mate was recorded. Following the first mating, females were then transferred to a new SY vial, and maintained for 48 h prior to the remating experiment; these vials were used to record offspring counts and determine male sterility. Males were considered sterile if there was no larval development from the vials in which their mates were housed after mating. Females who also produced no offspring after a remate were considered infertile and excluded from analyses. 48 h following their first mating, females were then transferred into a new vial containing a 5-d-old *st^1^*male, where female remating latency was recorded. Following the second mating, females were then transferred into two successive vials for 3 d each, so that only offspring laid after both matings were included in sperm competition analyses. Paternity was assigned based on eye-color markers, with *Dah* focal males and *st^1^* competitor males producing distinguishable offspring. For focal mate and remating experiments, a threshold of 4 min was used to exclude pseudomatings without sperm transfer. Experimental thresholds were set at 2.5 and 5 h, for focal mate and remate, respectively.

### Experiment 3: Dietary Consumption and Choice Experiments.

#### Food consumption.

We first measured dietary consumption using a Flypad automatic sip detection system (31). We measured dietary consumption of UM and FM males on protein- and/or cholesterol-rich diets used in experiment 2 (LPC, LPO, HPC, and HPO). For both P:C ratios we included both cholesterol-containing (0.3 g/L) (LPC and HPC) and cholesterol-absent (LPO and HPO) diets. We conducted assays over multiple experimental batches, in a fully factorial design. Males were maintained in cages on experimental diet for 10 d post eclosion, starved on water agar for 24 h, before cumulative sips were totaled. Sips were recorded for 30 mins (n = 728 males).

#### Food preference.

In a further set of experiments, we measured preference for protein- or cholesterol-rich diets (LPC, LPO, HPC, and HPO) in a fully factorial design. Males were communally housed in single- or mixed-sex environments and maintained for 3 to 5 d before being placed in experimental cages (n = ~100 males per cage), where they were provided with the choice of two diets in vials placed in separate grommets of the cage. Males localized to the media, and vial, were counted hourly. UM and FM were tested in separate experiments. UM males were assayed for 8 nonconsecutive days. FM males were measured for 3 consecutive days after their separation from females.

### Experiment 4: Female Mating Experiments.

Variation in female mating and remating rates on experimental diets in Experiments 1 to 3 could potentially confound direct effects of diet on male traits by generating variation in male mating rates. To examine whether this was a likely confound, we conducted two separate, complementary assays. In both assays, w^Dah^ females were used, to mimic experimental conditions for focal FM males in experiments 1 to 2. In the first assay, we quantified female mating latency, remating latency, and fecundity across diets. Virgin females were grown on SY, then collected and housed in vials on experimental diets (LPC, LPO, HPC, and HPO) for 10 d before being mated once with 3-d-old virgin *Dah* males (N = 897) over 3 batches, with latency to mate measured. Here, males were grown and maintained under SY conditions prior to their allocation to the experimental mating vial, ensuring female responses were not confounded by males. A portion of mated females (N = 245) were used to calculate female fecundity on experimental diets and another (N = 490) being used to measure remating latency. Females used for fecundity assays were drawn from the latency experiment to ensure that all individuals had mated prior to egg-laying measurements. Females were individually housed in vials on experimental diets (LPC, LPO, HPC, and HPO) and permitted to lay eggs for 24 h, before being counted. Since remating latency did not vary detectably across diets, we conducted a second assay to quantify cumulative remating over an extended period, testing whether dietary effects might instead emerge in repeated mating behavior. In this second assay, 2-d-old virgin females were housed with 2-d-old virgin *Dah* males in vials (N = 40) on experimental diets (LPC, LPO, HPC, and HPO) for 6 nonconsecutive days with matings recorded at half-hourly intervals (N = 90 intervals total). Additional vials were maintained and used to replenish dead flies.

### Statistical Analysis.

All analyses were performed in R Statistical Software v4.1.2 (32). Data wrangling was carried out using dplyr (33) and tidyverse (34), and figures were made using ggplot2 (35). Survival and latency analyses were performed using survival (36) and coxme (37) packages. Generalized linear models (GLMs) and generalized linear mixed models (GLMMs) were fitted using stats (38) and lme4 (39).

**Fig. 2.**
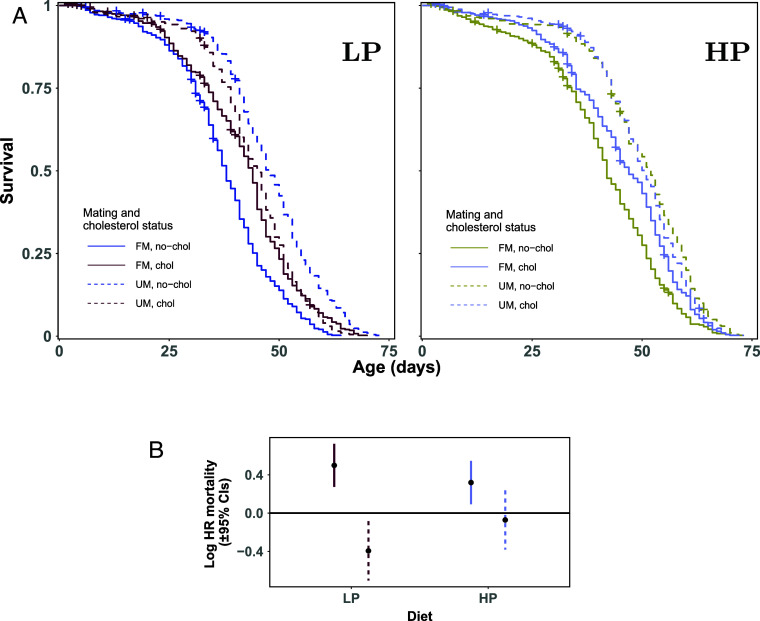
Dietary cholesterol depletion reduces lifespan in frequently mated males, but extends lifespan in unmated males on a low-protein diet, in a high-powered comparison. (*A*) Survival curves for males maintained on low-protein (LP; diet B3, Fig. 1*A*) and high-protein (HP; diet D3; Fig. 1*A*) diets under different mating (frequently mated, FM; unmated, UM) and cholesterol conditions. (*B*) Log hazard ratio mortality of the unique effect of dietary cholesterol deprivation in FM and UM males. Values are expressed relative to cholesterol-containing diets (baseline = 0), with positive values indicating increased mortality when cholesterol is omitted. N = 4,428 focal males; on average 500 to 600 per group.

For experiment 1, nutritional geometry (NG) lifespan analysis (Fig. 1*C*) was carried out using the linear model fixed-ratio diet approach outlined in (40). For comparison, optima P:C estimates were also computed using a quadratic approach in the OptimaRegion package (41). Apart from one optima region estimate (*SI Appendix*, Table S2) both approaches were concordant. Landscapes were fitted using a smoothing parameter of λ = 0.35, which determines the flexibility of the response surface. To estimate the marginal effect of cholesterol depletion (Fig. 1 *A* and *B*), Cox proportional hazards models were used. All latency traits were also analyzed using Cox proportional hazards models. This included male mating latency (*SI Appendix*, Fig. S2) and remating latency (*SI Appendix*, Fig. S4) in experiment 2, and female mating and remating latency in experiment 4 (*SI Appendix*, Fig. S6). For experiment 2 lifespan in demographic cages, survival was analyzed using Cox mixed-effects models with cage fitted as a random effect (Fig. 2).

**Fig. 3.**
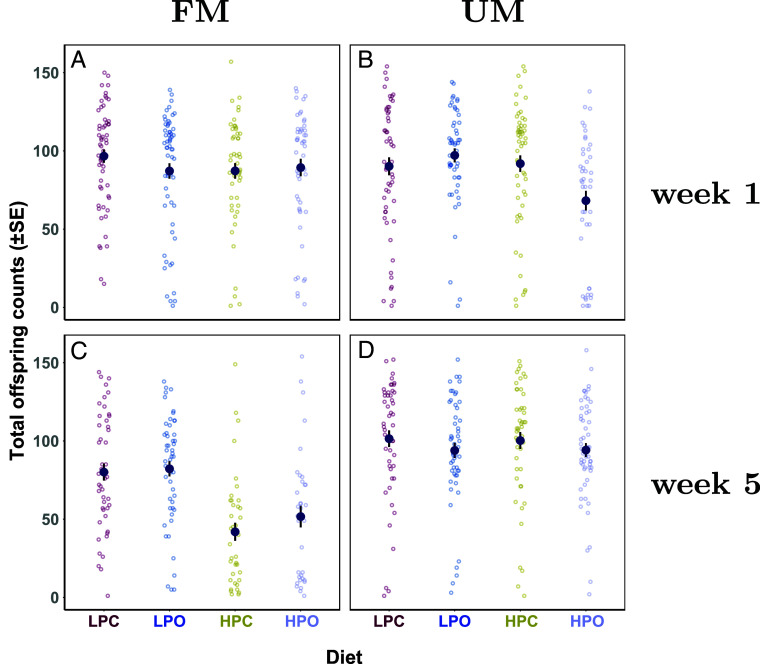
High-protein diet reduces offspring production primarily in older, frequently mated males. (*A*–*D*) Number of offspring sired by focal males under different dietary (low-protein, LP; high-protein, HP), cholesterol (present, C; absent, O), and mating (frequently mated, FM; unmated, UM) conditions. Panels correspond to combinations of mating and cholesterol conditions. Colored points represent the total number of offspring sired per male; black points indicate arithmetic means ± SE. N = 801 mates; N = 69,030 offspring.

**Fig. 4.**
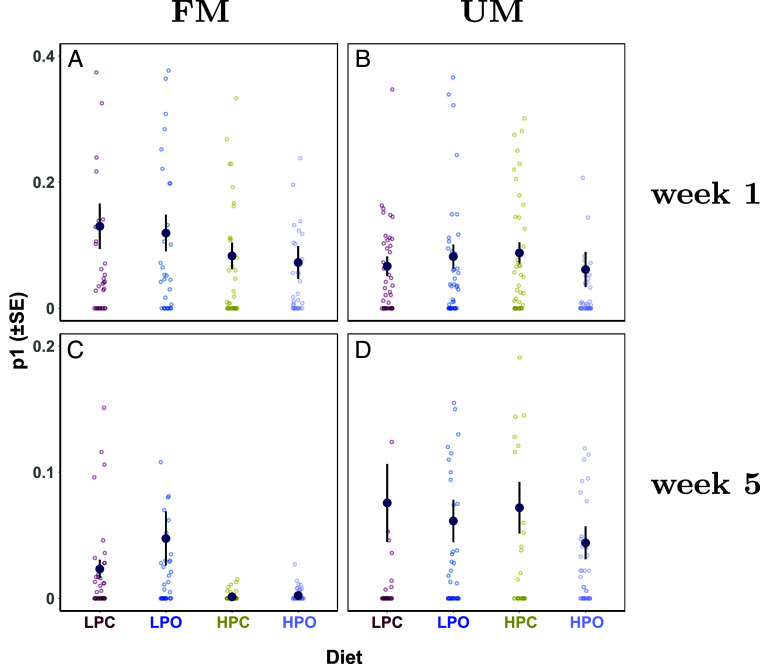
HP diet reduces focal male paternity proportion (p1) primarily in older, frequently mated males. (*A*–*D*) Paternity proportion (p1) of focal males under different dietary (low-protein, LP; high-protein, HP), cholesterol (present, C; absent, O), and mating (frequently mated, FM; unmated, UM) conditions. Panels correspond to combinations of mating and cholesterol conditions. Colored points represent proportion of offspring sired by focal males after females remated with a competitor *st^1^* male; black points indicate arithmetic means ± SE. N = 656 mates; N = 91,504 offspring.

For the remaining response variables, we fitted GLMs or GLMMs as appropriate to the structure of the data. These included offspring production (Fig. 3), sterility (*SI Appendix*, Fig. S3), paternity proportion (Fig. 4), dietary consumption (*SI Appendix*, Fig. S5), dietary choice (*SI Appendix*, Fig. S8), female fecundity (*SI Appendix*, Fig. S6), and female cumulative remating rate (*SI Appendix*, Fig. S7). Predictor structure depended on the experiment and response variable. For male reproductive traits in experiment 2, we tested each factor (cholesterol, P:C, mating status) in a full factorial model including all two- and three-way interactions. Where applicable, similar interaction structures were used in other analyses to allow decomposition of effects via marginal contrasts. For dietary choice assays in experiment 3, separate mixed models were fitted for the relevant contrasts. Communal housing was considered a random effect in addition to observation-level random effects to correct for overdispersion (42). Estimates were then bias-adjusted using the package emmeans (43). For experiment 4, female fecundity models consisted of P:C, cholesterol, and their interaction. Latency models for this experiment also included batch as an additive variable. Cumulative female remating rate (*SI Appendix*, Fig. S7) was analyzed using a binomial generalized linear mixed model, with the number of observed matings relative to the number of potential matings as the response, dietary P:C and cholesterol (and their interaction) as fixed effects, and vial identity included as a random effect to account for repeated measures.

(Partial) likelihood ratio (LR) chi-squared tests served as our primary inferential control for family-wise Type I error when assessing whether a factor influenced the response (either alone or in interaction). When the LR chi-squared test indicated a significant effect for a factor (significant coefficient and/or interactive term containing a coefficient), we decomposed that effect by estimating marginal effects (conditional contrasts) of the factor at each combination of the other two binary variables. These marginal effects are reported with estimated differences and SE to show where the effect lies and its direction. Because the LR chi-squared test was the primary inferential test, these marginal effects are presented as descriptive, post hoc decompositions to aid interpretation; the marginal-effect p-values are unadjusted and should not be treated as independent confirmatory tests. LR chi-squared tests were performed using a “type II” ANOVA; “type II” tests were chosen given the presence of interactive terms in the model, the respect for the principle of marginality, and their inferential relevance (44, 45). Given this, all LR chi-squared tests reported in the Results all have 1 degree of freedom.

The error distribution and link function used for each GLM or GLMM are specified in the corresponding supplementary tables, together with the exact model structure for each response. Quasi-distributions were used to correct for overdispersion where necessary. Since reproductive assays of 1-wk and 5-wk-old males were carried out at different times, these data have been analyzed separately, and direct comparisons of ages have not been performed. To prevent the inclusion of sterile males from biasing offspring counts, they have been omitted from the analysis, but retained for analysis of sterility and paternity proportion. With one exception (reported in results) no qualitative differences in interpretation arise from their inclusion.

## Results

### Lifespan Optima Occur at Relatively High Protein-to-Carbohydrate Ratios (Around 1:2; Experiment 1).

We first examined the relationship between lifespan and diet using a nutritional geometry (NG) approach, in our solid, chemically defined (holidic) medium. Twelve diets, spanning four protein-to-carbohydrate ratios (P:C) and four nutrient densities (Fig. 1*A*) were used to provide coverage over the area of the nutritional landscape relevant to male fitness (18, 19, 29). We compared frequently mated (FM) and unmated (UM) males with and without dietary cholesterol (chol; no-chol) to detail any topographical differences between these conditions. These landscapes were estimated using fixed ratios, rather than measured nutrient intakes.

Estimated lifespan optima ranged from 1:2.5 in FM males deprived of cholesterol, to 1:1.2 in FM males provided with cholesterol (UM cholesterol 1:2.3 [CI 1:3.2 to 1:1.8]; FM cholesterol 1:1.2 [CI 1:1.5 to 1:1]; UM no cholesterol 1:2.3 [CI 1:2.9 to 1:1.9]; FM no cholesterol 1:2.5 [CI 1:3.4 to 1:2]; Fig. 1*C* and *SI Appendix*, Table S2). These estimates are substantially higher than those reported from previous work carried out using capillary-feeder (CAFE) assays, which tend toward 1:16 (7, 19, 46).

### Cholesterol Depletion Curtails Lifespan in Mating Males (Experiments 1 and 2).

Removing cholesterol—our sole source of dietary sterols—from adult males across our NG diets (experiment 1), resulted in a marked reduction in lifespan in frequently mated (FM) males. The magnitude of this effect increased with both protein-to-carbohydrate ratio (P:C), and nutrient concentration of the diet (Fig. 1 *A* and *B*). We had hypothesized that sterol intake postmaturation is required largely for reproductive investment, and any dietary sterol deficiency may lead to the depletion of somatic sterol reserves, with costs realized as reduced lifespan under sustained mating. Consistent with this idea, the increase in mortality observed was only present when males were permitted to mate freely throughout their lifespan.

To examine this effect in more detail, and to test whether sterol depletion results in male reproductive costs, we conducted a higher powered comparison (experiment 2) between a relatively high P:C diet (D3, 1:1; HP - high-protein diet) where cholesterol depletion had precipitated an exacerbation of mortality in experiment 1, and a relatively low P:C diet (B3, 1:6; LP - low-protein diet) where no mortality exacerbation was observed. Here, we detected effects of P:C (χ^2^ = 35.24, *P* < 0.001), mating status (χ^2^ = 61.12, *P* < 0.001), and cholesterol depletion (χ^2^ = 8.1, *P* = 0.0044; Fig. 2) on lifespan.

In concordance with the results of experiment 1, in all but one group (unmated, cholesterol-depleted males), HP resulted in longer lifespans relative to LP (*SI Appendix*, Table S3). The effect of cholesterol depletion was contingent upon mating status (χ^2^ = 35.24, *P* < 0.001). FM males in both LP and HP experienced increased mortality (LP: HR = 1.65, *P* < 0.001; HP: HR = 1.38, *P* = 0.0058; Fig. 2 and *SI Appendix*, Table S4). In contrast, we found that cholesterol depletion extended lifespan in UM males fed LP (HR = 0.67, *P* = 0.013; Fig. 2 and *SI Appendix*, Table S4), which suggests a reduced target intake of sterols in the UM state, and a cost of cholesterol overconsumption. Differences in the magnitude of cholesterol effects between experiments 1 and 2 likely reflect differences in experimental set-up (vials *versus* demographic cages), which may influence baseline mortality rates and the expression of diet-dependent effects.

### Modest Dietary Effects On Reproduction in Young Males (Experiment 2).

We tested multiple components of male reproduction under conditions of sterol depletion, in both young (1-wk-old), and old (5-wk-old) males (Figs. 3 and 4 and *SI Appendix*, Figs. S2–S4). In young males we found no effect of protein-to-carbohydrate ratio (P:C), mating status, or cholesterol depletion on latency to mate (*SI Appendix*, Fig. S2 *A* and *B*). We observed a small but significant effect of P:C on male sterility (χ^2^ = 4.28, *P* = 0.038; *SI Appendix*, Fig. S3 *A* and *B*), in which we were unable to reject the null within a marginal effects analysis (*SI Appendix*, Table S5). Note, males producing no offspring were classified as sterile and excluded from offspring count analyses, but retained for analyses of sterility and paternity (see Materials & Methods). Both P:C, and the three-way interaction between P:C, mating status and cholesterol depletion, had significant effects on the number of offspring sired from this mating (χ^2^ = 4.47, *P* = 0.034; χ^2^ = 8.82, *P* = 0.003; Fig. 3 *A* and *B*). The three-way interaction was primarily driven by the relatively low number of offspring sired by unmated (UM) males, fed a cholesterol-depleted HP diet (*SI Appendix*, Table S6).

We detected no significant impact of P:C, or cholesterol depletion on focal male paternity proportion (Fig. 4 *A* and *B*), a measure of postcopulatory reproductive success. However, we found that focal male dietary P:C modulated remating latency of females, following mating with focal males (χ^2^ = 7.52, *P* = 0.0061; *SI Appendix*, Fig. S4 *A* and *B*), with remating rates tending to be higher under high-protein (HP) diets, particularly in the UM cholesterol-depleted treatment (*SI Appendix*, Table S7). This pattern suggests a reduction in the ability of males to induce sexual refractoriness in their mates due to HP diet. This effect, however, may have been driven by the inclusion of sterile males (excluding sterile males: χ^2^ = 3.65, *P* = 0.056).

### Pre- and Postcopulatory Success Are Higher On LP Than On HP in Aged, Mating Males (Experiment 2).

Our expectation was that both protein-to-carbohydrate ratio (P:C) and cholesterol depletion would have larger effects in old males, due to the cumulative effect of nutrient imbalance. Similarly, we expected frequently mated (FM) males to show stronger dietary responses—either due to the continual replenishment of sperm and seminal fluid proteins (47) on which flies are reliant on nutrients from food for their synthesis, or because of the increased metabolic costs associated with mating, further accelerating aging in suboptimal dietary conditions (48, 49).

In line with this, we found that dietary macronutrient ratio significantly affected multiple components of pre- and postcopulatory male reproductive performance in 5-wk-old males, except for latency to mate. There were significant effects of dietary P:C in the proportion of sterile focal males (χ^2^ = 10.96, *P* < 0.001; *SI Appendix*, Fig. S3 *C* and *D*), number of offspring sired (χ^2^ = 16.13, *P* < 0.001; Fig. 3 *C* and *D*), remating latency of the mated female (χ^2^ = 17.06, *P* < 0.001; *SI Appendix*, Fig. S4 *C* and *D*), and paternity proportion (χ^2^ = 6.63, *P* = 0.01; Fig. 4 *C* and *D*).

All of these P:C effects were contingent upon male mating treatment, with stronger effects in FM males and weak or no effects in unmated (UM) males: for sterility (χ^2^ = 8.87, *P* = 0.0029; *SI Appendix*, Fig. S3 *C* and *D*), number of offspring sired (χ^2^ = 28.9, *P* < 0.001; Fig. 3 *C* and *D*), remating latency of the mated female (χ^2^ = 8.44, *P* = 0.0037; *SI Appendix*, Fig. S4 *C* and *D*), and paternity proportion (χ^2^ = 13.21, *P* < 0.001; Fig. 4 *C* and *D*).

Directionally consistent with previous studies (18, 19), we found the effects of high-protein diet (HP) diet to be deleterious to both pre- and postcopulatory success in FM males (*SI Appendix*, Table S8–S11). For sterility and paternity proportion, these effects were dependent on dietary cholesterol availability. HP reduced cholesterol-depleted male fertility (coef = −2.46, SE = 0.65, *P* < 0.001; *SI Appendix*, Table S8) and paternity proportion (coef = -3.13, SE = 1.35, *P* = 0.021; *SI Appendix*, Table S9). This corresponds to an > 11-fold increase in the odds of sterility, and > 22-fold reduction in the odds of focal paternity compared to LP diet.

In contrast, the effects of dietary P:C on offspring production and remating latency were independent of cholesterol. HP reduced the number of offspring sired (chol: coef = −0.65, SE = 0.12, *P* < 0.001; no chol: coef = −0.46, SE = 0.12, *P* < 0.001; *SI Appendix*, Table S10) and increased female remating rate (chol: HR = 1.93, SE = 0.2, *P* = 0.0011; no chol: HR = 2.29, SE = 0.21, *P* < 0.001; *SI Appendix*, Table S11). This corresponds to approximately 40% fewer offspring and ~2-fold increase in remating rate, relative to LP.

Together, these results show that high-protein diets reduce reproductive performance in frequently mated males, despite being associated with longer lifespan under most conditions, indicating a nutritional trade-off between lifespan and reproduction.

### Sterol Depletion Comes at Negligible Cost to Reproduction in Young and Old Males (Experiment 2).

While the depletion of dietary sterols induced costs to lifespan in actively reproducing males, we identified no consistent effects on reproductive traits. Specifically, sterol depletion did not affect mating latency, sterility, or paternity proportion in either young or old males.

For female remating latency, we detected a small mating-dependent effect of cholesterol in 5-wk-old males (χ^2^ = 3.88, *P* = 0.049) driven by overall trends for higher remating rates in cholesterol-depleted unmated (UM) males and lower remating rate in sterol depleted frequently mated (FM) males (*SI Appendix*, Fig. S4). However, there were no significant differences between treatments in the marginal effects analyses of cholesterol depletion on remating latency (*SI Appendix*, Table S12).

With the exception of number of offspring sired by 1-wk-old UM males (*SI Appendix*, Table S6), sterol depletion conferred no observable cost to reproduction. In contrast, HP diets reduced multiple aspects of reproductive success, in old, FM males. Together, these results indicate that the primary costs of sterol depletion are realized through lifespan, with limited effects on reproduction.

### Costs of Cholesterol Depletion and HP Are Not Driven by Differences in Consumption (Experiment 3) or Female Remating Rate (Experiment 4).

We recognized two potential confounds which could alter the interpretation of cholesterol depletion’s costs to male lifespan, or high-protein (HP) costs to male reproductive success. First, nutrient intake can vary widely, dependent on macronutrient content of provided media (50, 51). One possibility therefore is that underconsumption could induce a dietary restriction-like response. Using the FlyPad, we measured feeding behavior in 1-wk-old males (experiment 3). We detected a small increase in sip count for unmated (UM) males relative to frequently mated (FM) males (χ^2^ = 7.9, *P* = 0.0049; *SI Appendix*, Fig. S5). Consumption of cholesterol-depleted media was contingent upon diet and mating status (χ^2^ = 11.62, *P* < 0.001; χ^2^ = 5.1, *P* = 0.023; *SI Appendix*, Fig. S5). However, given the modest differences in mean consumption (range: 156 to 278 sips), the disparate direction of the marginal effects of cholesterol-depletion (*SI Appendix*, Table S13), and that HP effects are caused almost entirely by low sip counts in unmated, cholesterol-depleted males (*SI Appendix*, Table S14), it is unlikely that differences in consumption are a primary driver of either cholesterol costs to lifespan, or HP costs to reproductive success.

Second, we tested whether diet-dependent changes in female behavior could confound male mating-dependent effects (experiment 4). Female fecundity and remating frequency can be modulated by macronutrient availability (52–54), and—for fecundity—sterol availability (14). If female remating rate varied with diet, this would in turn impact male mating frequency in the FM treatment. Hence, diet-induced effects on females could indirectly alter male investment in reproduction, confounding the direct effect of diet treatments on males. However, we found that previously unmated females maintained on our experimental diets displayed no difference in their frequency of mating, or remating (*SI Appendix*, Fig. S6 *A* and *B*), despite reduced fecundity due to low-protein diet (χ^2^ = 17.07, *P* < 0.001) and cholesterol depletion (χ^2^ = 11.11, *P* < 0.001; *SI Appendix*, Fig. S6C and Tables S15 and S16). To test whether dietary effects might instead emerge in repeated mating behavior—in line with conditions females experience housed with frequently mated males—we measured cumulative remating rates through spot sampling (*SI Appendix*, Fig. S7). Surprisingly, we found neither P:C, nor cholesterol content of the diet affected remating rate (χ^2^ = 2.33, *P* = 0.13; χ^2^ = 1.53, *P* = 0.22). Although visual trends suggest reduced cumulative remating under cholesterol-depleted HP conditions, these differences were not statistically supported. Together, these results suggest that the deleterious effects of cholesterol-depletion on lifespan, and of HP on male reproductive success, are unlikely to be explained by differences in nutrient intake or female remating behavior.

### Male Food Choice Indicates a Preference Toward Maximizing Reproductive Success Over Longevity (Experiment 3).

We then tested our males’ dietary preference between low- and high-protein diets. Classical evolutionary theory predicts that when nutrient resources cannot simultaneously maximize both somatic maintenance and reproductive output, selection will favor allocation (or feeding strategies) that prioritize reproduction at the expense of the soma (55). This principle is supported by NG studies in females, showing that animals often choose diets that enhance reproductive output even when these shorten lifespan (7, 56). Consistent with this, we found that frequently mated (FM) males exhibited a preference toward the low-protein diet (LP; the diet which maximizes reproductive success), the strength of which did not significantly vary with cholesterol presence or absence (coef = 0.41, SE = 0.12, *P* < 0.001; *SI Appendix*, Fig. S8*C*). Unmated (UM) males exhibited a similarly strong preference for LP, again independent of cholesterol availability (coef = 0.44, SE = 0.097, *P* < 0.001; *SI Appendix*, Fig. S8*D*). Thus, preference for LP was independent of mating status, despite the fact only FM males experience reproductive costs of high-protein (HP) diets. This could indicate that UM males, held in single-sex groups, lack the plasticity to respond differently to mating males (46), possibly because of the irregularity of isolation from females.

We further examined the capacity of our males to exhibit preferences based on dietary cholesterol content. UM males showed a significant preference for cholesterol-free food in an HP background but not a LP background (coef = -0.26, SE = 0.09, *P* = 0.0036; *SI Appendix*, Fig. S8*B*), whereas FM males displayed no preference (*SI Appendix*, Fig. S8*A*). This constitutes further evidence that the cholesterol concentration used was higher than optimal for nonreproductively active males (i.e. UM), and circumstantially suggests that FM males do not appear to integrate their internal cholesterol status into dietary decision-making, and thus fail to increase their preference for cholesterol-rich food despite elevated reproductive demands.

## Discussion

In this series of experiments, we carried out protein-to-carbohydrate (P:C) manipulations and sterol deprivation in male *D. melanogaster* under varying reproductive contexts, to detail the extent of effects on lifespan, and an array of reproductive measures. Our key findings are that cholesterol deprivation is realized as a somatic—but not reproductive—cost, and in a mating-dependent manner: Only males exposed to females suffer a lifespan penalty. While we interpret this as evidence that sterol-dependent costs are realized under sustained mating effort, it is important to note that the frequently mated and unmated treatments differ not only in mating activity but also in broader aspects of the social environment. Males housed with females may experience differences in behavior, activity, or physiological state, which could also influence sensitivity to dietary sterol availability. Our study applies nutritional geometry (NG) in males using an exome-matched amino acid ratio in a fully holidic, solid diet that does not result in unusually short lifespan. We find that male lifespan is optimized at a much higher P:C than previously estimated. We additionally find that late-life male precopulatory and postcopulatory reproductive success is maximized under lower rather than higher P:C, broadly in line with previous work (18, 19). We also find that, in choice tests, in line with females (7), males bias feeding toward macronutrient ratios that promote reproductive performance rather than lifespan; males are also capable to discriminating food with and without cholesterol. Finally, our data show that female fecundity and remating responses to diet can be decoupled, and, surprisingly, female remating rate is invariant to dietary cholesterol and P:C.

Animals are expected to moderate intake of available nutrients in line with nutritional targets, but targets are susceptible to change, and depend ultimately on broad suite of variables (4, 56). For example, sex (19), olfactory cues (57), temperature (58), and—putatively—the life-history strategy pursued by the consumer can alter target intakes (measured as preferences) in macro- and micronutrient consumption (7). Historically, the cost of male reproduction within life-history research has been overlooked, despite sex interacting with diet when mediating traits (59). This has likely arisen due to the traditional idea that male reproduction is “cheap” relative to female reproduction (i.e. small sperm *versus* large eggs), in addition to the difficulty in assaying male reproductive success, relative to the comparative ease of measuring female reproduction (i.e. counting eggs or offspring). Nonetheless, reproductive investment in males constitutes a cost, both in terms of energy, and molecular components (20, 60). Since unmated males invest less in ejaculates, relative to frequently mated counterparts (47), target intakes for seminal nutritional components, such as cholesterol, should theoretically be lower. We see some evidence of this: Unmated males on a low-protein diet live longer on cholesterol-free diet than on a cholesterol-containing diet (Fig. 2), which is consistent with the idea that too much dietary cholesterol is harmful in the absence of reproduction. Similarly, we found that males were capable of sensing the presence of dietary cholesterol and detected a measurable preference in unmated males away from cholesterol-containing diet in a high-protein background (*SI Appendix*, Fig. S8). Together, these may indicate that the cholesterol concentration in our high-protein diet (where males invest less heavily in reproduction) exceeds the target intake for nonreproducing males.

Crucially, we find males deprived of dietary cholesterol suffer a somatic cost—one which is induced exclusively when permitted to mate over their whole life—indicating a cost associated with reproduction in a nutrient-depleted state (Figs. 1*C* and 2). In addition, nondirect reproductive effects associated with housing environment, such as increased locomotor activity, courtship effort, or social interactions, may also elevate physiological demand and thereby amplify the consequences of sterol limitation. One possibility for the mechanism underlying this phenomenon, suggested by (13) in relation to female *Drosophila*, is the cumulative effect of sequestering sterols from somatic tissue for reallocation to meet reproductive needs. Sterols are abundant within ejaculates (61), and organisms are predicted to prioritize reproductive output over somatic maintenance (5). Moreover, sterols are a vital component of the ejaculate. For example, male lady beetles (*Coccinella septempunctata*) suffer complete spermatogenic failure, becoming sterile, when deficient in phytosterols or cholesterol (62). One prediction of this model is that costs of sterol depletion rise in accordance with reproductive investment. While we do not measure male reproductive investment per se, we did find that the best P:C for male reproductive performance is lower than that for lifespan. If male reproductive performance reflects reproductive investment, then diets that maximize reproduction should incur the greatest costs under sterol depletion. Instead, we observe the opposite pattern: The effect of sterol depletion weakens as diets become more carbohydrate-biased (Fig. 1*B*) and is absent in our higher-powered comparison of high- and low-protein diets (Fig. 2). Alternatively, variation in male investment in reproduction (e.g., making sperm and seminal fluid) across nutritional landscapes, might not be reflected in reproductive performance, if for example, nonoptimal nutrition diminishes male reproductive success while not alleviating the costs of reproductive investment, or if males compensate for poor condition with higher investment in ejaculate production. Note that the difference in effect sizes of cholesterol depletion on lifespan observed between the NG and direct comparison experiments may be due to the difference in experimental housing. The direct comparison experiment used cages which, while maximizing the number of flies which can be maintained per vial of food, impose a larger force of extrinsic mortality relative to vials, since flies need to maintain high motility to feed. If sterol depletion increases risk of death principally in frail individuals, then this effect will be attenuated in cages, which could explain the smaller effect sizes in the direct comparison experiment relative to the NG experiment. More generally, these observations emphasize that housing conditions can influence multiple aspects of male physiology beyond mating itself, and may therefore contribute to variation in the magnitude of sterol-dependent lifespan effects.

A further prediction of the sterol sequestering model is the potential for reproductive success to improve under conditions of cholesterol abundance. If cholesterol is a primary limiting nutrient in ejaculates, providing it in excess might be expected to free up more energetic resources to meet reproductive output. As such, unmated males on a cholesterol-containing diet might be expected to have elevated reproductive performance because, throughout their life, they do not lose sterols via frequent mating, but they are able to consume them and allocate them to other bodily functions. However, we did not observe this: Unmated males on cholesterol diets did not have elevated reproductive performance; conversely cholesterol resulted in reduced lifespan on a low-protein diet, suggestive of a somatic cost to cholesterol overconsumption (Figs. 3–4 and *SI Appendix*, Figs. S2–S4). One explanation for our results overall may be that the costs of underconsumption or overconsumption of cholesterol are borne exclusively by the soma—impacting lifespan—possibly acting alongside protein overconsumption as a synergistic effect. Deleterious effects of protein intake on female fly lifespan are well detailed (63, 64), but there is no evidence of costs of cholesterol overconsumption to date (13). Recent work has demonstrated systemic cholesterol induces an upregulation in TOR activation (65), and thus growth, providing a putative mechanistic role for the relationship between increased dietary cholesterol and mortality. Alternatively, sterol depletion may interact with reproductive costs to induce somatic costs if, for example, the metabolic stress of reproduction requires an increase in recycling of sterols present in cell membranes. Cholesterol is also integral in the biosynthesis of a key arthropod ecdysteroid—ecdysone (66)—and its dysregulation via under- or overconsumption may be the proximate cause of somatic costs.

Extensive previous work on the topography of the lifespan nutritional landscape in both female and male *D. melanogaster* has identified relatively well-defined optima at low P:C ratios (~1:16 to 1:6; see ref. 46). With few exceptions (18), NG studies have used capillary feeder (CAFE) assays with either holidic or yeast-based media. Our findings differ from these in two main respects: 1) we estimate male lifespan optima at approximately 1:2 P:C, independent of cholesterol or mating status, and 2) our lifespan surface is broader, with a flatter topography overall (Fig. 1*C*). There are several possible explanations for these discrepancies. Biologically, CAFE systems have been shown to constrain both lifespan and reproduction relative to solid media. This may result from mechanical difficulties in feeding from capillaries, altered olfactory or tactile cues, or the unnatural feeding posture required. Likewise, studies using meridic (semidefined) solid diets have also reported reduced lifespan (18), suggesting that the mechanisms underlying shortened lifespans across systems are diverse and nonoverlapping. Consequently, optima derived from CAFE-based studies may not fully capture the NG of flies feeding freely on solid substrates. Note, however, that Lee *et al*., did recapitulate P:C dietary lifespan optima under solid food diets, where lifespan was not unusually short. Some evidence exists of suboptimal male lifespan on carbohydrate-biased diets, in one of the few studies to perform dietary experiments on holidic, solid media (12). Together, these findings may point to fundamental differences in the composition or metabolism of holidic versus yeast-based diets, potentially reflecting the high bioavailability of holidic nutrients (21).

In addition to these biological factors, two experimental features of our study may also contribute to the observed differences. First, our nutritional landscapes were generated using the fixed P:C ratios of the formulated diets, rather than those adjusted for actual intake. As such, our response surfaces reflect responses mapped to diet composition rather than consumption, which could influence the apparent breadth of the optima. Second, our nutrient concentrations were lower than those typically used in comparable studies. We note, however, that this difference does not necessarily indicate a methodological mismatch: CAFE assays require exceptionally high nutrient concentrations to sustain adequate feeding, whereas our solid-food system does not. Finally, our diets are exome-matched with respect to the amino acid ratios within the protein constituent of the diet. Much of the focus of nutritional targets remains at the macronutrient level, but targets equally apply to micronutrients (21). Individuals on fixed diets consuming suboptimal nutrient ratios will be compelled to compromise on nutrient intake, with the degree of over- or underconsumption of any nutrient contingent upon nutrient sensing and rules of compromise (67). Given this, the cost of high protein consumption may be attenuated if the relative amino acid abundance is proportional to relative targets, as it theoretically is when amino acid ratios are exome matched (11).

Our data show that while female fecundity is reduced under sterol or protein deprivation, as expected (7, 13), perhaps surprisingly female remating rate is invariant to dietary cholesterol and P:C ratio (*SI Appendix*, Figs. S6 and S7). While female fecundity and remating are decouplable, via genetic alterations to females (68, 69) or to the male ejaculate (70), the fecundity and refractoriness responses of intact females to intact males typically occur in parallel. Females respond to male seminal signals after mating (71) and the responses are known to be dependent on food levels: Both remating and egg production increase with yeast levels (54). Our finding that females reduced fecundity but not remating when either protein or cholesterol was reduced might be explained by insufficient or invariant yeast odors in our holidic diets. Work by (72) shows that the nutritional content of the food interacts with female detection of yeast-specific odors to modulate receptivity to mating. Both stimuli are required to elevate female receptivity, and the receptivity and fecundity responses can be uncoupled with targeted dietary manipulations. While all our holidic diet treatments did contain both acetic acid and amino acids, it is possible that the concentrations of either or both nutrients were insufficient to modulate female receptivity responses. For example, we used 3 g/L acetic acid consistently across all diets, as opposed to >10 g/L in (72). Using a constant low level of acetic acid across all recipes might limit variation in female remating rate, even when amino acid concentrations change. Regardless, our results indicate that the impacts of diet on male lifespan and reproductive traits were not confounded by female remating rates, as we found no evidence of female remating rates varying.

## Conclusions

Our study demonstrates that sterol deprivation imposes a somatic cost on reproductively active male *D. melanogaster*, manifesting as reduced lifespan without affecting reproductive success. Our findings also reveal that male flies exhibit a nutritional trade-off between high- and low-protein diets, with optimal lifespan achieved at higher protein-to-carbohydrate ratios than previously reported. The data underscore the importance of considering both macronutrient and micronutrient interactions in dietary studies, and suggest that the nutritional costs of male reproduction should receive greater attention. Future research should investigate the effects of other sterol species, for example, sterols that are less readily converted into steroid hormones than cholesterol (15), to explore mechanisms underpinning the costs and benefits of sterol consumption. Testing the impact of dietary sterols on males that genetically lack the ability to make sperm and/or seminal fluid (73, 74) will likely be informative, and could reveal which aspects of male reproduction are most sterol demanding. Similarly, disentangling the relative contributions of mating effort and the broader sociosexual environment will be an important direction for future work.

## Supplementary Material

Appendix 01 (PDF)

Dataset S01 (XLSX)

## Data Availability

All data supporting this manuscript are available via Dryad (10.5061/dryad.q573n5tx9) (75).
