# Supplementary material for: Mating-dependent lifespan cost of sterol depletion in male Drosophila melanogaster
Source: Proc Natl Acad Sci U S A. 2026 Jun 2;123(23):e2533735123. doi: 10.1073/pnas.2533735123 (PMC13250600; doi:10.1073/pnas.2533735123)
Supplement: Supplementary file 1 — Appendix 01 (PDF) [file pnas.2533735123.sapp.pdf]

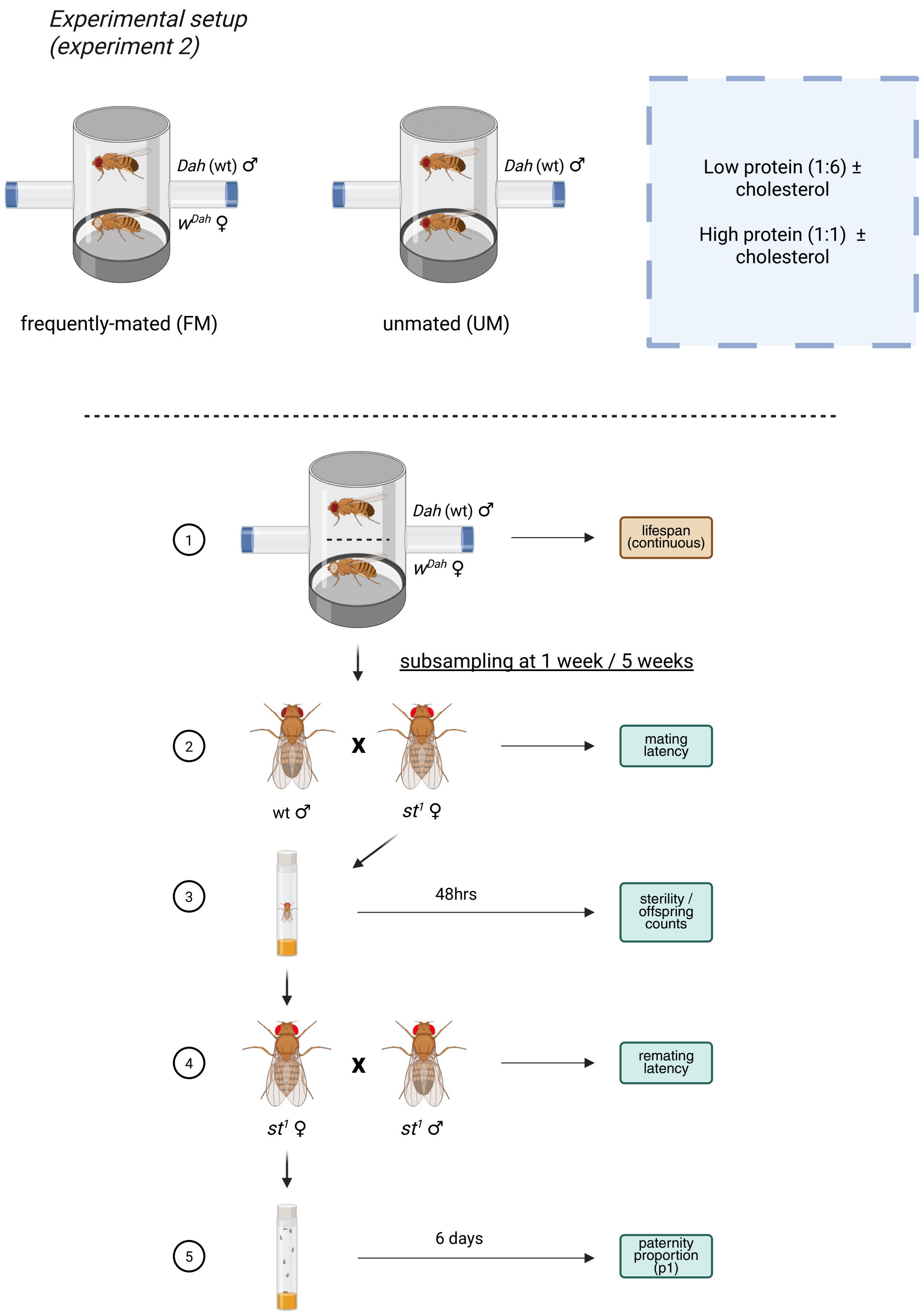

Fig. S1. Experimental schematic for experiment 2. Males were maintained under fully factorial combinations of diet (low-protein [1:6] or high-protein [1:1], each with or without cholesterol) and

mating environment (frequently mated [FM] or unmated [UM]) in demography cages. Lifespan was recorded continuously in cages (1). Subsets of males were removed at 1 and 5 weeks of age and individually housed on their experimental diet for 3 days prior to reproductive assays. Males were first paired with virgin *st¹* females to measure mating latency (2). Following mating, females were transferred to oviposition vials for 48 h to quantify offspring production and male sterility (3). Females were then paired with *st¹* males to measure remating latency (4), before being transferred across sequential vials to assess paternity proportion (p1) based on offspring eye colour (5). Males removed from lifespan cages for reproductive assays were right-censored in survival analyses. Created with BioRender.com.

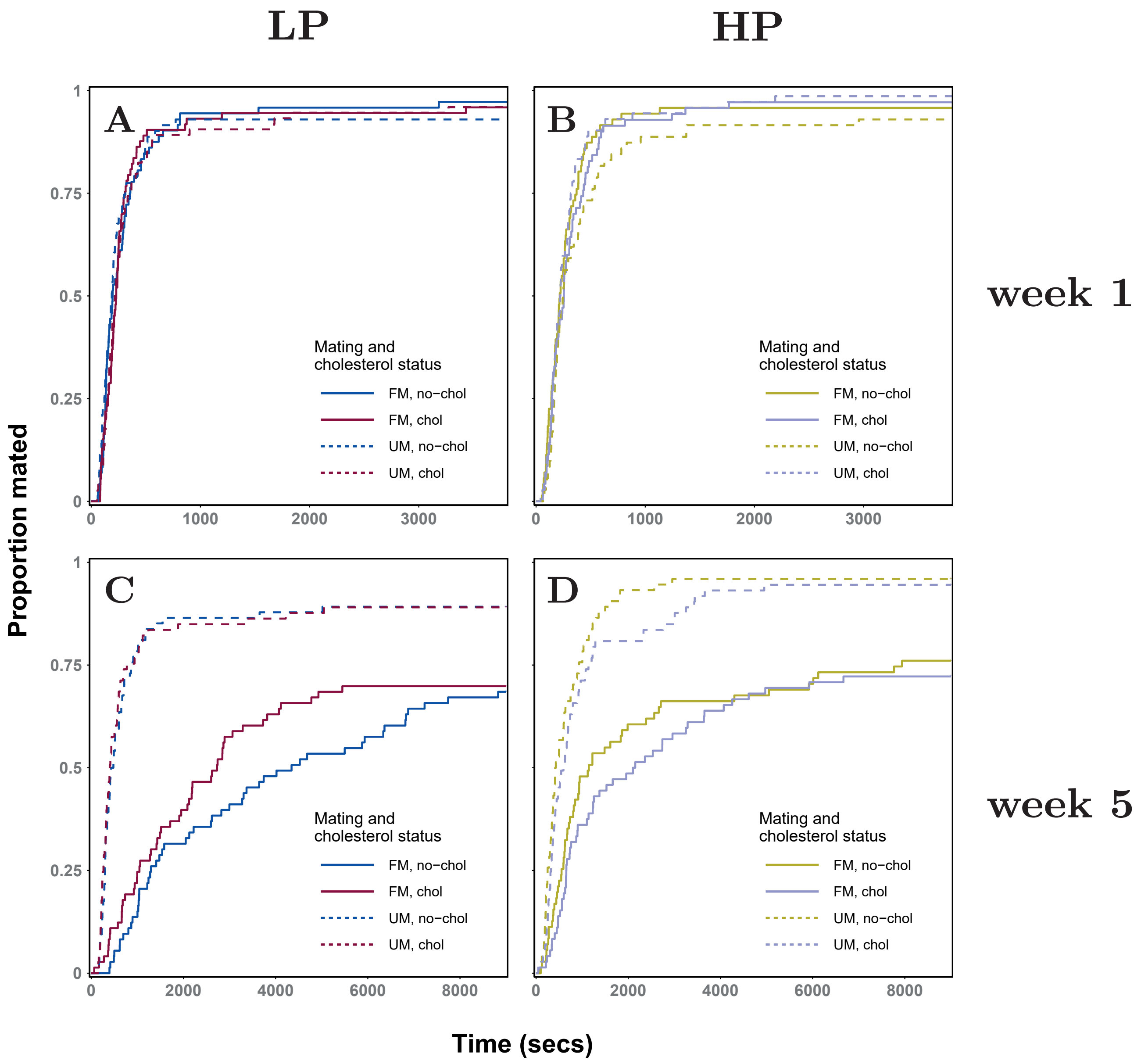

Fig. S2. Older males are slower to mate. (A–D) Cumulative proportion of focal males mating over time with 3-day old virgin *st¹* females under different dietary (low-protein, LP; high-protein, HP), mating (frequently-mated, FM; unmated, UM) and cholesterol conditions. N = 1,169 mate pairs.

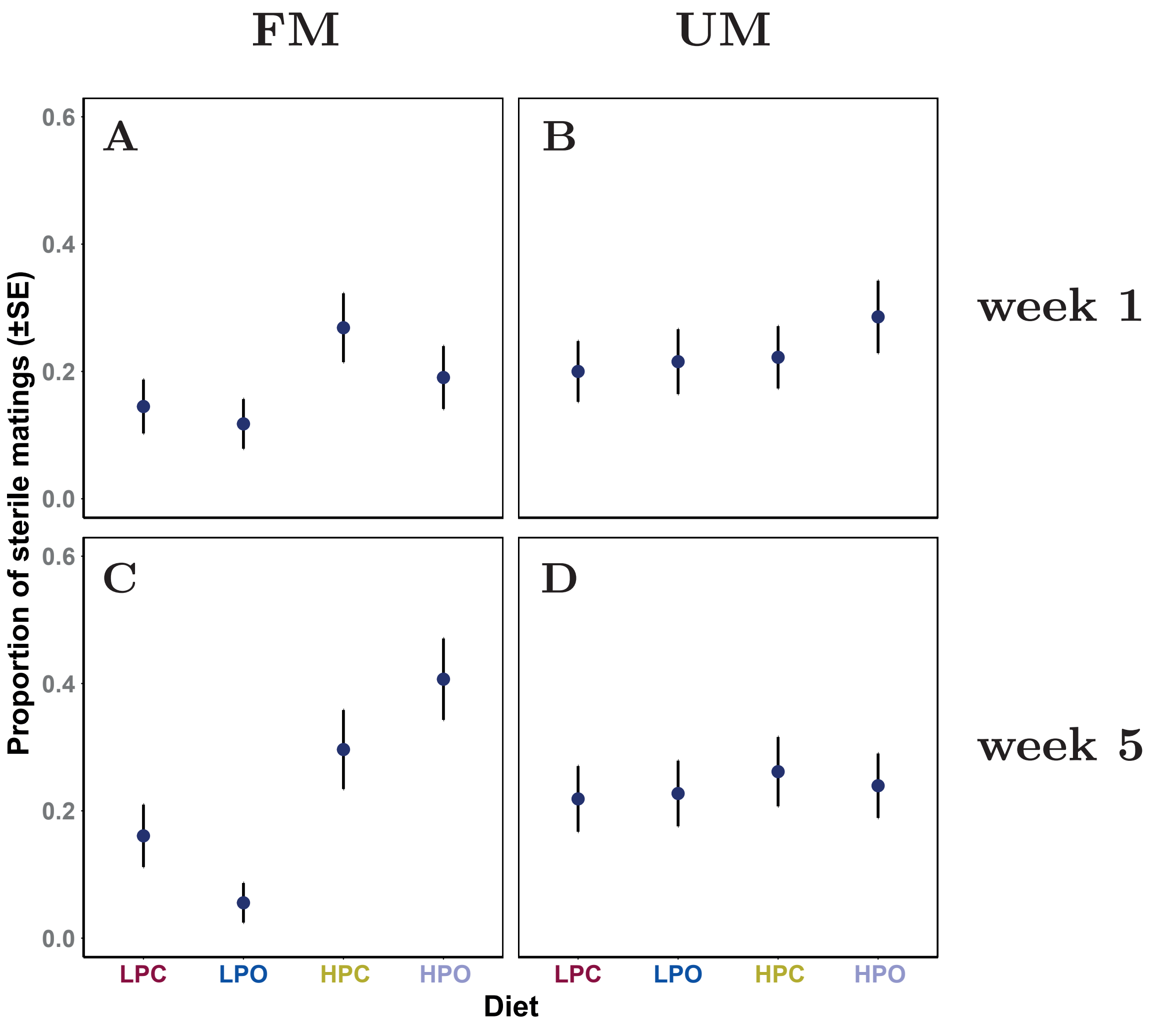

Fig. S3. High-protein diet increases sterility primarily in older, frequently-mated males. Proportion of males producing no offspring after a successful mating under different dietary (low-protein, LP; high-protein, HP), cholesterol (present, C; absent, O), and mating (frequently-mated, FM; unmated, UM) conditions. N = 1,026 mates; N = 225 mate events without offspring.

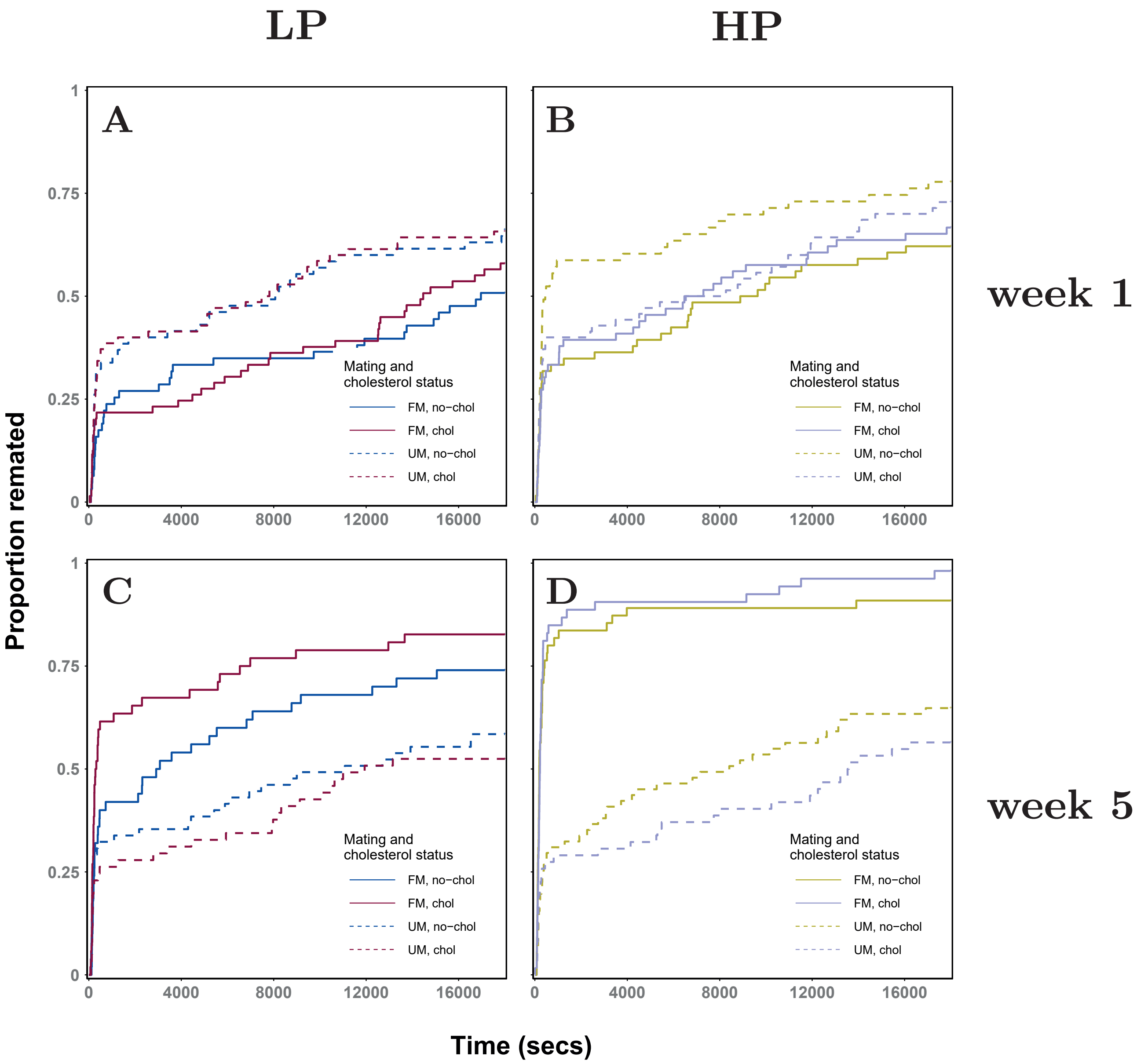

Fig. S4. Female remating after initial mating to focal males varies with focal male diet, with no consistent effect of cholesterol depletion. Female remating rate represents an inverse measure of focal male post-copulatory success, with higher remating indicating lower fitness. (A–D) Cumulative proportion of females remating over time with 5-day old virgin *st*[1] males, 48h after initial mating with focal males. Treatments correspond to different focal male dietary (low-protein, LP; high-protein, HP), mating (frequently-mated, FM; unmated, UM) and cholesterol conditions. N = 1,001 mate pairs.

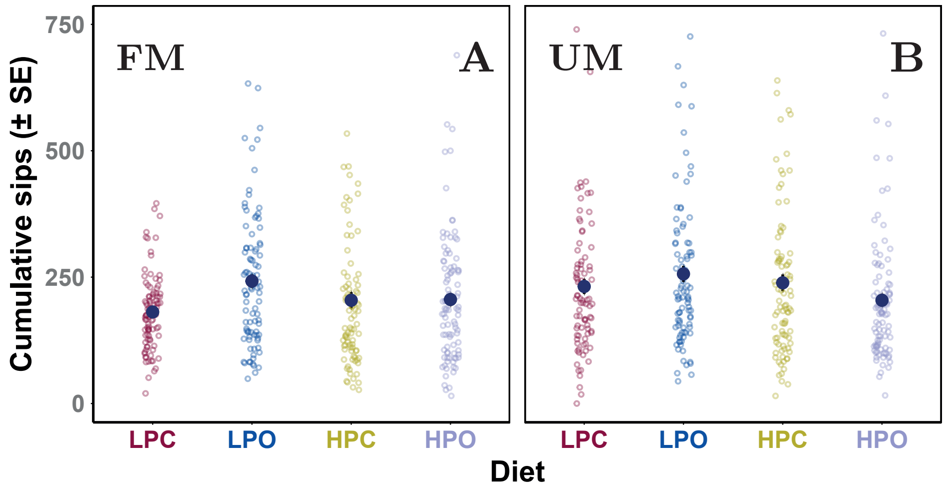

Fig. S5. No evidence compensatory feeding driving deleterious effects of high-protein diet or cholesterol deprivation in males. (A–B) Cumulative sips of media from focal males measured on FlyPad over a 30-minute period under different dietary (low-protein, LP; high-protein, HP), cholesterol (present, C; absent, O), and mating (frequently-mated, FM; unmated, UM) conditions. Panels correspond to mating treatments, with (A) FM and (B) UM males. Coloured points represent cumulative sip count per male; black points indicate arithmetic means ± SE. Males were assayed at 1-week-old. N = 728 males across 8 experimental batches.

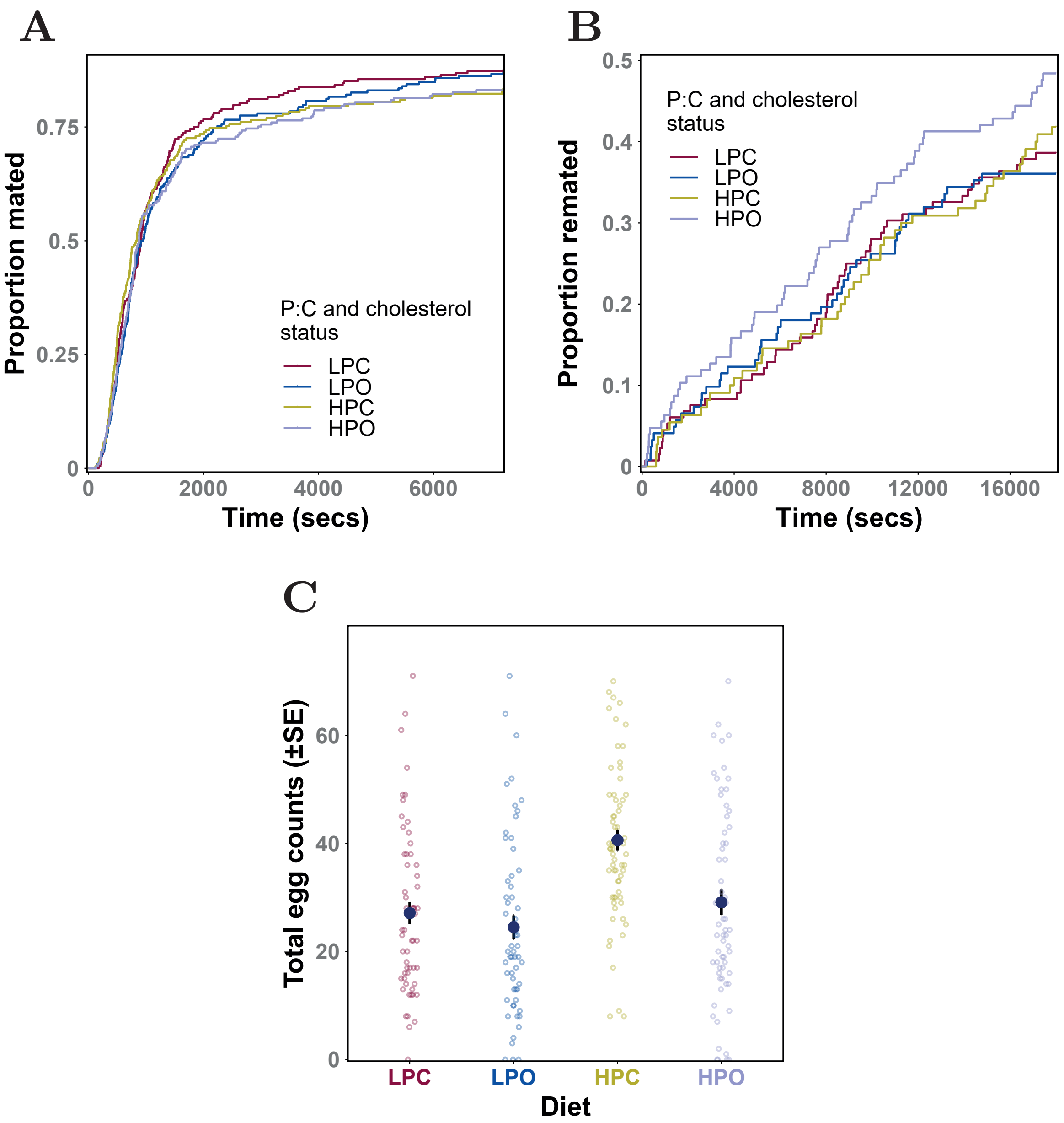

Fig. S6. No evidence of female mating or remating rate driving deleterious effects of high-protein diet or cholesterol deprivation on males via disparate costs of mating. (A–B) Latency to mate (A) and remate (B) of 1-week old females maintained on experimental diets, showing no detectable effect of dietary (low-protein, LP; high-protein, HP) or cholesterol (present, C; absent, O) conditions. (C) Female fecundity (of females which successfully mated) under the same dietary conditions, showing reduced fecundity under LP and cholesterol depletion. Coloured points represent number of eggs laid in 24h per female; black points indicate arithmetic means ± SE. w^{Dah} females used to replicate experimental conditions under which focal frequently-mated males were kept. N = 897 mate pairs for mating latency; N = 490 mate pairs for remating latency; N = 245 females, 7,492 eggs for fecundity assay.

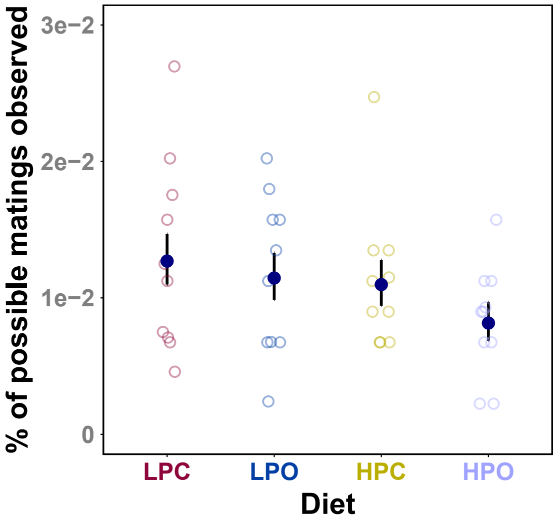

Fig. S7. No evidence that cumulative female remating rates drive effects of high-protein diet or cholesterol deprivation in males via disparate costs of mating. Remating rates of females under different dietary (low-protein, LP; high-protein, HP) and cholesterol (present, C; absent, O) conditions. Coloured points represent the cumulative number of matings observed in a vial divided by the total number of potential matings within that vial, across the experiment. Black points represent model estimates ± 68% CIs. w^{Dah} females used to replicate experimental conditions under which focal frequently-mated males were kept. On average, 5 males and 10 females were present per vial. N = 40 vials; N = 90 sampling intervals.

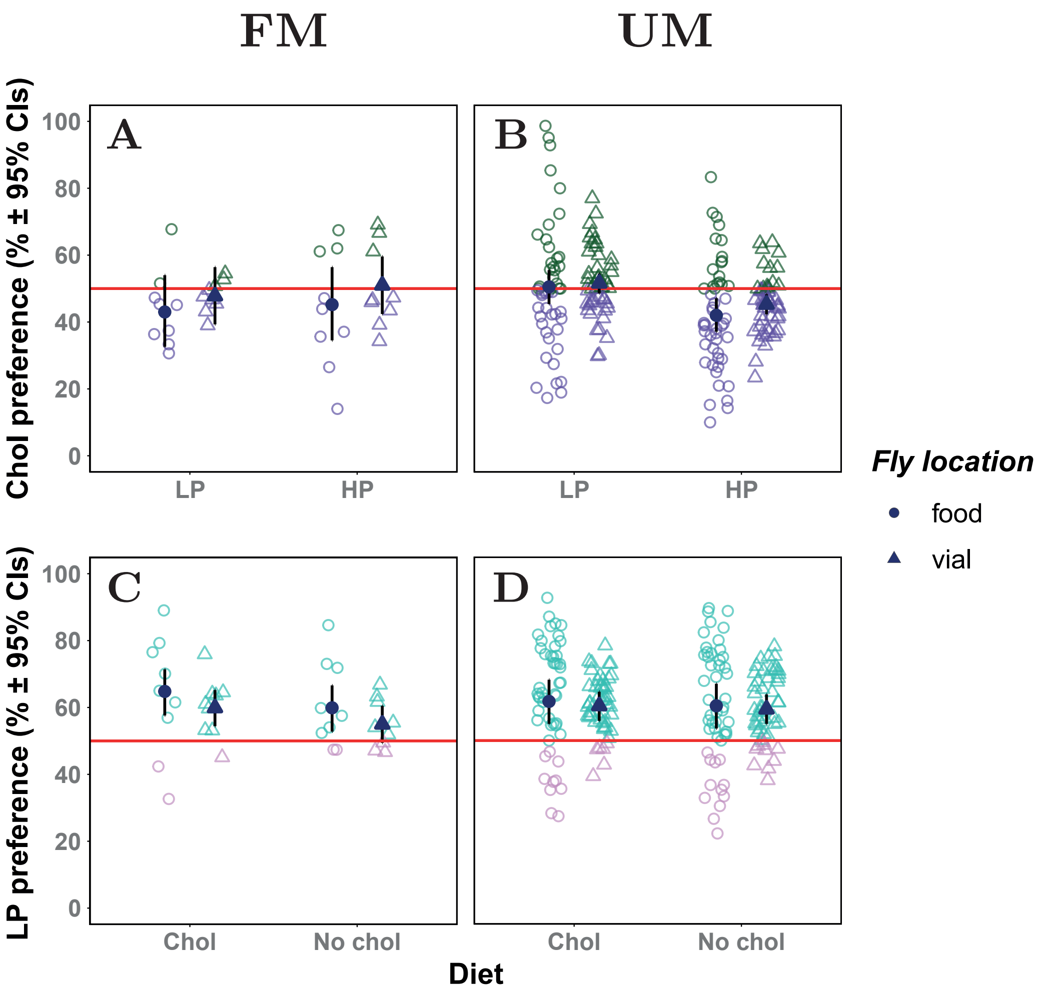

Fig. S8. Males can detect the presence of cholesterol in media, and feed preferentially on cholesterol depleted media, when unmated and fed high-protein diet. Males consistently prefer low-protein food regardless of mating status or cholesterol availability. FM = frequently-mated; UM = unmated; LP = low-protein diet; HP = high-protein diet. (A–B) Preference for cholesterol-containing media when *Dah* males were presented with a choice between cholesterol-containing and cholesterol-depleted media, shown for FM (A) and UM (B) males in LP and HP dietary backgrounds. (C–D) Preference for LP media when males were presented with a choice between LP and HP media, shown for FM (C) and UM (D) males in cholesterol-containing and cholesterol-depleted backgrounds. Within each panel, coloured points represent cage-day proportions above or below the null expectation of 50%, and shapes denote fly location (circles = food; triangles = vial). Black points show model-estimated means ± 95% CIs. Flies scored as being on food represent a subset of flies within a vial; thus, individuals scored as on food were also included in counts within vial. FM and UM experiments were carried out separately. For UM, N = 20 cages; N = 70 intervals. For FM, N = 12 cages; N = 21 intervals. N = ~120 males per cage for UM experiment; N = ~40 males and 80 females per cage for FM experiment.

Table S1. Summary of previous nutritional geometry studies estimating lifespan and reproductive optima in *Drosophila melanogaster*. Studies are compared by sex, experimental style, adult diet type, early-life mating treatment, and whether adults were maintained in a single-sex environment. Reported values indicate the optimal lifespan and reproductive output identified in each study, with units retained from the original publications.

	**Sex**	**Experiment style**	**Adult Diet**	**Early-life mating**	**Single-sex environment**		**Optimal lifespan**	**Optimal reproduction**
**Lee *et al.*, 2008**	F	CAFE	hydrolyzed yeast	24 hrs	Y		22 days	5 eggs / day
**Jensen *et al.*, 2015**	F	CAFE	meridic	virgin	Y		25.13 ± 2.13 days	1.24 ± 0.23 eggs / day
	M						26.13 ± 1.17 days	8.48 ± 1.55 offspring / day
**Carey *et al.*, 2022**	F	solid	meridic	virgin; mated every 5 days thereafter	Y		36.80 ± 2.65 days	53.74 ± 3.4 eggs / mate
	M						34.35 ± 2.22 days	17.87 ± 2.96 offspring / mate

Table S2. Comparison of estimated P:C lifespan optima in experiment 1 using the Morimoto *et al.* and del Castillo *et al.* approaches (Fig. 1C). Lower and upper confidence intervals (LCI and UCI) represent 2.5 and 97.5 percentiles of model predicted observations.

**TREATMENT**	**Morimoto**	**del Castillo**	**Morimoto LCI**	**Morimoto UCI**
CHOL FM	1: 1.2	2.7: 1	1: 1.5	1: 1
CHOL UM	1: 2.3	1: 2.8	1: 3.2	1: 1.8
NOCHOL FM	1: 2.5	1: 3.1	1: 3.4	1: 2
NOCHOL UM	1 :2.3	1: 2.5	1: 2.9	1: 1.9

Table S3. Marginal effects of high-protein diet on male lifespan in experiment 2 (Fig. 2). Low-protein diet reference. Cox mixed-effects model.
Model: lifespan ~ P:C * cholesterol * mating status + (1|cage)

**TREATMENT**	**COEF**	**HAZARD RATIO**	**SE**	**P**
CHOL FM	-0.31663416	0.7285973	0.1155881	0.0062
CHOL UM	-0.52209953	0.5932736	0.1581480	0.001
NOCHOL FM	-0.4961551	0.6088672	0.1147758	<0.0001
NOCHOL UM (NS)	-0.1993724	0.8192448	0.1583712	0.21

Table S4. Marginal effects of cholesterol deprivation on male lifespan in experiment 2 (Fig. 2).
Cholesterol-containing diet reference. Cox mixed-effects model.
Model: lifespan ~ P:C * cholesterol * mating status + (1|cage)

TREATMENT	COEF	HAZARD RATIO	SE	P
FM LP	0.49855060	1.6463334	0.1147684	<0.0001
FM HP	0.3190296	1.3757921	0.1157390	0.0058
UM LP	-0.39445131	0.6740498	0.1583620	0.013
UM HP (NS)	-0.07172413	0.9307876	0.1581981	0.65

Table S5. Marginal effects of high-protein diet on sterility in 1-week-old males in experiment 2 (Fig. S3).
Low-protein diet reference. Binomial GLM. Logit link.
Model: larval development ~ P:C * cholesterol * mating status
NB sterility modelled as presence of viable offspring.
Note, no significant marginal effects despite significant chi-squared test.

TREATMENT	COEF	SE	P
CHOL FM (NS)	-0.7735	0.4392	0.078
CHOL UM (NS)	-0.13353	0.41188	0.75
NOCHOL FM (NS)	-0.5680	0.4946	0.25
NOCHOL UM (NS)	-0.37648	0.41087	0.36

Table S6. Marginal effects of cholesterol deprivation on offspring counts in 1-week-old males in experiment 2 (Fig. 3).
Cholesterol-containing diet reference. QuasiPoisson GLM. Log link.
Model: offspring counts ~ P:C * cholesterol * mating status

TREATMENT	COEF	SE	P
CHOL FM (NS)	-0.10237	0.08159	0.21
CHOL UM (NS)	0.01884	0.07989	0.81
NOCHOL FM (NS)	0.02477	0.08171	0.76
NOCHOL UM	-0.35395	0.09328	0.0002

Table S7. Marginal effects of high-protein diet on female remating latency 48hrs after mating with 1-week-old focal males in experiment 2 (Fig. S4).
Low-protein diet reference. Cox proportional hazards model. Sterile males included.
Model: remating ~ P:C * cholesterol * mating status
Note, no significant marginal effects despite significant chi-squared test.

TREATMENT	COEF	HAZARD RATIO	SE	P
CHOL FM (NS)	0.32301	1.38128	0.21493	0.13
CHOL UM (NS)	0.15614	1.16899	0.20153	0.95
NOCHOL FM (NS)	0.29054	1.33715	0.22845	0.20
NOCHOL UM (NS)	0.40624	1.50116	0.20817	0.051

Table S8. Marginal effects of high-protein diet on male sterility in 5-week-old males in experiment 2 (Fig. S3).

Low-protein diet reference. Binomial GLM. Logit link.

Model: larval development ~ P:C * cholesterol * mating status

NB sterility modelled as presence of viable offspring.

TREATMENT	COEF	SE	P
CHOL FM (NS)	-0.7879	0.4703	0.094
CHOL UM (NS)	-0.23498	0.41362	0.57
NOCHOL FM	-2.4559	0.6505	0.0002
NOCHOL UM (NS)	-0.06800	0.40450	0.87

Table S9. Marginal effects of high-protein diet on paternity proportion (p1) in 5-week-old males in experiment 2 (Fig. 4).

Low-protein diet reference. Quasibinomial GLM. Logit link.

Model: larval development ~ P:C * cholesterol * mating status

TREATMENT	COEF	SE	P
CHOL FM (NS)	-2.9997	1.6590	0.072
CHOL UM (NS)	0.1082	0.3899	0.78
NOCHOL FM	-3.1271	1.3518	0.021
NOCHOL UM (NS)	-0.4389	0.4254	0.30

Table S10. Marginal effects of high-protein diet on offspring counts in 5-week-old males in experiment 2 (Fig. 3).

Low-protein diet reference. QuasiPoisson GLM. Log link.

Model: offspring counts ~ P:C * cholesterol * mating status

TREATMENT	COEF	SE	P
CHOL FM	-0.64783	0.12403	<0.0001
CHOL UM (NS)	-0.01179	0.08351	0.89
NOCHOL FM	-0.46472	0.11676	0.0001
NOCHOL UM (NS)	0.002397	0.083557	0.97

Table S11. Marginal effects of high-protein diet on female remating latency 48hrs after mating with 5-week-old focal males in experiment 2 (Fig. S4).

Low-protein diet reference. Cox proportional hazards model.

Model: remating ~ P:C * cholesterol * mating status

TREATMENT	COEF	HAZARD RATIO	SE	P
CHOL FM	0.6592	1.9333	0.2032	0.0011
CHOL UM (NS)	0.08512	1.08884	0.24102	0.72
NOCHOL FM	0.8266	2.2855	0.2139	0.0001
NOCHOL UM (NS)	0.12251	1.13033	0.21928	0.58

Table S12. Marginal effects of cholesterol deprivation on female remating latency 48hrs after mating with 5-week-old males in experiment 2 (Fig. S4).

Cholesterol-containing diet reference. Cox proportional hazards model.

Model: remating ~ P:C * cholesterol * mating status

Note, no significant marginal effects despite significant chi-squared test.

TREATMENT	COEF	HAZARD RATIO	SE	P
FM LP (NS)	-0.3553	0.7010	0.2178	0.10
FM HP (NS)	-0.1879	0.8287	0.1966	0.34
UM LP (NS)	0.14496	1.15599	0.23796	0.54
UM HP (NS)	0.18235	1.20003	0.22264	0.41

Table S13. Marginal effects of cholesterol deprivation on food consumption in 1-week-old males in experiment 3 (Fig. S5).

Cholesterol-containing diet reference. QuasiPoisson GLM. Log link.

Model: sips ~ P:C * cholesterol * mating status + batch

TREATMENT	COEF	SE	P
FM LP	0.28616	0.08016	0.0004
FM HP (NS)	0.004661	0.080674	0.95
UM LP (NS)	0.09478	0.07489	0.21
UM HP	-0.15858	0.07777	0.042

Table S14. Marginal effects of high-protein diet on food consumption in 1-week-old males in experiment 3 (Fig. S5).

Low-protein diet reference. QuasiPoisson GLM. Log link.

Model: sips ~ P:C * cholesterol * mating status + batch

TREATMENT	COEF	SE	P
CHOL FM (NS)	0.13038	0.08377	0.12
CHOL UM (NS)	0.03762	0.07661	0.62
NOCHOL FM	-0.15112	0.07693	0.05
NOCHOL UM	-0.21574	0.07609	0.0047

Table S15. Marginal effects of high-protein diet on female fecundity in ~ 2-week-old females in experiment 4 (Fig. S6).

Low-protein diet reference. QuasiPoisson GLM. Log link.

Model: eggs ~ P:C * cholesterol

TREATMENT	COEF	SE	P
CHOL	0.40301	0.09922	0.0001
NOCHOL (NS)	0.17273	0.10859	0.11

Table S16. Marginal effects of cholesterol deprivation on female fecundity in ~ 2-week-old females in experiment 4 (Fig. S6).

Cholesterol-containing diet reference. QuasiPoisson GLM. Log link.

Model: eggs ~ P:C * cholesterol

TREATMENT	COEF	SE	P
LP (NS)	-0.10205	0.11335	0.37
HP	-0.33233	0.09374	0.0005
